# Biofilm-associated healthcare-associated infections: mechanisms, clinical burden, and emerging therapeutic frontiers – a narrative review

**DOI:** 10.3205/dgkh000675

**Published:** 2026-09-10

**Authors:** Gargee Anand, Rijhul Lahariya

**Affiliations:** 1 All India Institute of Medical Sciences, Department of Microbiology, Patna, Bihar, India; 2 All India Institute of Medical Sciences, Patna, Bihar, India

**Keywords:** antimicrobial stewardship, central venous catheter, urinary catheters, endotracheal tubes, prosthetic implants, mortality

## Abstract

Biofilms are highly organized microbial communities embedded within an extracellular polymeric matrix and represent a critical yet underappreciated driver of healthcare-associated infections (HAIs). Their ability to colonize medical devices and host tissues underlies major infections, including central line-associated bloodstream infections, catheter-associated urinary tract infections, ventilator-associated pneumonia, and surgical site infections.

Biofilm formation is dynamic, multistage process involving attachment, maturation, and dispersal of microorganisms, each phase reinforcing persistence and therapeutic failure. The matrix impedes immune clearance and antimicrobial penetration, while quorum sensing, metabolic heterogeneity, and persister cell formation further enhance tolerance. Clinically important bacterial and fungal biofilms, particularly those formed by *Staphylococcus aureus*, *Pseudomonas aeruginosa*, and Candida species, exhibit resistance far exceeding planktonic cells. Diagnostic limitations obscure true disease burden, and current strategies relying on antimicrobials and device removal remain inadequate amid rising resistance.

This manuscript provides comprehensive synthesis of existing and novel strategies to mitigate HAIs, with particular emphasis on translational relevance.

## Abstract (de)

Biofilme sind hochorganisierte mikrobielle Gemeinschaften, die in eine extrazelluläre Polymermatrix eingebettet sind und einen kritischen, aber unterschätzten Auslöser nosokomialer Infektionen (NI) darstellen. Ihre Fähigkeit, Devices und Wirtsgewebe zu besiedeln, ist Ursache für schwere Infektionen, darunter ZVK-assoziierte Blutstrominfektionen, Katheter-assoziierte Harnwegsinfektionen, Beatmungs-assoziierte Pneumonien und postoperative Wundinfektionen.

Die Bildung von Biofilmen ist ein dynamischer, mehrstufiger Prozess, der Anlagerung, Reifung und Ausbreitung von Mikroorganismen umfasst, wobei jede Phase die Persistenz und das Therapieversagen verstärkt. Die Matrix behindert die Immunabwehr und die antimikrobielle Penetration, während Quorum Sensing, metabolische Heterogenität und Persistenzzellbildung die Toleranz erhöhen. Klinisch wichtige bakterielle und pilzliche Biofilme, insbesondere solche, die von *Staphylococcus aureus*, *Pseudomonas aeruginosa* und Candida-Arten gebildet werden, weisen eine Resistenz auf, die weit über die von Planktonzellen hinausgeht. Diagnostische Einschränkungen verschleiern die tatsächliche Krankheitslast, und aktuelle Strategien, die auf antimikrobiellen Mitteln und der Entfernung von Devices basieren, bleiben angesichts zunehmender Resistenzen unzureichend.

Das Manuskript bietet eine Zusammenführung bestehender und neuer Strategien zur Reduzierung von NI mit besonderem Schwerpunkt auf der translationalen Relevanz.

## Introduction

Healthcare-associated infections (HAIs) remain among the most frequent and devastating complications in modern medicine, representing a major global public health challenge [1]. Microbial biofilms underlie the majority of device-associated infections, are implicated in up to 70% of all microbial diseases and represent a major driver of HAIs [2]. Once established on indwelling medical devices such as central venous catheters (CVCs), urinary catheters, endotracheal tubes (ET), and prosthetic implants, biofilms form a highly resistant microbial reservoir that is nearly impossible to eradicate with standard antibiotic regimens [3], [4], [5], [6], [7]. This persistent mode of infection not only complicates clinical management but also accelerates the development and dissemination of antimicrobial resistance (AMR), with an estimated annual economic burden exceeding USD 5 trillion [8], [9]. 

With the increasing use of invasive medical devices in contemporary healthcare, the incidence of biofilm-associated infections has escalated globally [10]. Virtually all implanted or indwelling devices are susceptible to microbial colonization, turning life-saving interventions into potential sources of chronic infection [4]. Biofilm-associated infections now account for the majority of persistent bacterial infections in humans and are implicated in nearly half of all HAIs linked to medical devices [11].

Understanding biofilms is essential to tackling HAIs, as nearly 80% of chronic infections are biofilm-associated [12]. Their inherent resistance to antibiotics fuels persistent infections and the rise of multidrug-resistant pathogens, projected to cause 10 million deaths annually by 2050 [13]. However, current infection control and treatment strategies, largely designed for planktonic bacteria, fail to adequately address biofilm-associated infections, particularly on medical devices and sterile sites [14], [15], [16]. The interplay between HAIs and biofilms represents a silent yet escalating threat to patient safety and healthcare systems worldwide. This review seeks to close the critical gap between microbiological insight and clinical practice by translating the complex biology of biofilms into actionable strategies for the prevention, diagnosis, and treatment of HAIs. Addressing this challenge will require not only improved infection prevention protocols and device stewardship but also urgent innovation in anti-biofilm strategies and antimicrobial development.

## Method

A narrative review was conducted to evaluate the role of biofilms in HAIs, AMR, and emerging anti-biofilm strategies. Relevant articles were identified through search of PubMed using keywords such as “biofilm”, “healthcare-associated infections”, “device-associated infections”, “antimicrobial resistance”, “quorum sensing”, and “anti-biofilm therapy”. Original research articles, reviews, clinical studies, and guideline documents published in English were included. The retrieved literature was screened for relevance, and eligible studies were critically reviewed and synthesized to provide an overview of biofilm biology, clinical significance, diagnostic challenges, and current and emerging management approaches.

## Results

### The biofilm life cycle: a dynamic survival strategy of pathogens

Biofilms represent a unifying pathogenic strategy across bacterial and fungal pathogens implicated in HAIs [10]. They enable microbial persistence on indwelling medical devices, hospital surfaces, and host tissues, thereby driving chronicity, therapeutic failure, and recurrence [17]. Unlike planktonic organisms, biofilm-embedded pathogens display recalcitrance, a coordinated, multicellular phenotype of tolerance to antibiotics even at high concentrations and immune evasion that is central to the epidemiology of ventilator-associated pneumonia (VAP), catheter-associated urinary tract infection (CAUTI), central line-associated bloodstream infection (CLABSI) and periprosthetic joint infections (PJI) [18].

#### Formation and maturation of bacterial biofilm

Bacterial biofilm development (Figure 1 (Fig. 1)) follows a stepwise progression:

- Initial attachment (reversible docking): Microbes transiently adhere to host or device surfaces through weak physicochemical interactions – hydrophobic forces, van der Waals attractions, and electrostatic charges, guided by environmental cues and bacterial motility [19].
- Stable adhesion (irreversible locking): Bacterial surface structures such as pili, fimbriae, and adhesins anchor the cells firmly. This molecular “handshake” triggers the secretion of extracellular polymeric substances (EPS), marking the transition from a free-living to a sessile lifestyle [19].
- Biofilm maturation (structural and functional expansion): Rapid bacterial replication, EPS accumulation, and spatial organization form complex three-dimensional communities with nutrient channels and protective architecture. These mature biofilms resist immune clearance and antimicrobial penetration [19].
- Dispersal (seeding of new niches): Environmental and quorum-sensing (QS) signals activate biofilm dispersal, releasing planktonic cells to colonize new surfaces. Dispersal may occur via erosion (gradual shedding), sloughing (bulk release), or active seeding (from within) [19].

#### Quorum sensing – the molecular orchestration of bacterial biofilms 

Biofilm formation is not a random bacterial aggregation but a highly regulated, multicellular behaviour, coordinated through QS, a chemical communication system that enables bacteria to sense and respond to population density [20]. As bacterial numbers rise, diffusible signaling molecules, such as N-acyl homoserine lactones in Gram-negative species, autoinducing peptides in Gram-positives, and the interspecies autoinducer-2, accumulate in the extracellular environment [20]. Once a critical threshold is reached, these molecules trigger synchronized gene expression across the community. QS regulates both biofilm maturation and dispersal, facilitating colonization of new niches [20]. In clinical settings, this network contributes to persistent device-associated infections (DAI) and therapeutic failure. However, most mechanistic insights into QS are derived from controlled in vitro systems, and the complexity of polymicrobial interactions along with host immune factors in vivo may significantly alter QS dynamics, thereby limiting the translational applicability of these findings to real-world infection settings [21].

#### The extracellular matrix (ECM) – architect and shield of the bacterial biofilm

EPS is the biochemical framework that transforms bacterial aggregates into resilient biofilms. Composed of polysaccharides, proteins, lipids, and extracellular DNA, it anchors cells, maintains structural integrity, and protects against environmental stress [22]. EPS facilitates nutrient retention, immune evasion, and antibiotic tolerance by limiting drug penetration and host defenses [22]. It also promotes QS and horizontal gene transfer, enhancing antimicrobial resistance. Thus, EPS functions as both the structural scaffold and protective barrier underlying biofilm persistence [22]. Nevertheless, the relative contribution of EPS-mediated resistance versus metabolic dormancy in clinical infections remains debated, as antibiotics can penetrate biofilms yet fail due to physiological heterogeneity [23], [24].

#### Candida biofilms: a parallel fungal survival strategy

Fungal pathogens, particularly Candida species, mirror bacterial biofilm strategies but with following unique features (Figure 2 (Fig. 2)) relevant to HAIs:

- Initial adhesion: Yeast cells rapidly adhere to biotic or abiotic surfaces, including mucosal epithelia and medical implants. This initial contact is mediated by adhesins, cell wall mannoproteins, and electrostatic interactions [25].
- Early biofilm development: Adherent yeast cells proliferate to form a basal monolayer. Hyphal transformation begins, a critical virulence trait, accompanied by the secretion of an ECM, rich in β-glucans, proteins, and extracellular DNA [25].
- Maturation: The biofilm architecture becomes increasingly complex, with dense networks of yeast, hyphae, and pseudo hyphae encased in ECM. Water channels form within the matrix, facilitating nutrient flow and metabolic cooperation. Antifungal resistance escalates sharply at this stage [25].
- Dispersal: Mature biofilms release yeast cells or hyphal fragments into the bloodstream or surrounding tissues. These disseminated cells exhibit an enhanced capacity to colonize new niches, contributing to recurrent and invasive candidiasis [25].

#### Quorum sensing in Candida – a driver of biofilm resilience

In *Candida (C.) albicans*, QS orchestrates biofilm development and drug resistance through population-dependent signaling [26]. The QS molecule farnesol inhibits hyphal formation by repressing hypha-associated genes (TUP1, CRK1) and upregulating efflux transporters (CDR1, PDR16), tipping the balance toward a biofilm-embedded phenotype [26]. Histidine kinase CHK1 mediates farnesol responsiveness, with CHK1 mutants forming biofilms despite QS inhibition [26]. Farnesol also contributes to biofilm dispersal under nutrient stress, enabling colonization of new sites. QS may regulate surface adhesion via anti-adhesins like YWP1, dampening biofilm adherence in response to population cues [26].

#### Extracellular matrix – the structural and protective backbone of Candida biofilms

In *C. albicans*, the ECM forms a dense, polymeric shield critical to biofilm architecture and defense. Composed of proteins, chitin, extracellular DNA, and β-1,3-glucans, this matrix stabilizes the biofilm’s complex 3D structure, spanning 50 to 350 µm, and anchors cells against shear forces and antifungal insults [26]. Beyond physical integrity, the ECM serves as a chemical fortress, impeding immune cell access and sequestering antifungal drugs, thus enabling persistent infection [26].

### Molecular basis of biofilm-mediated resistance

Three interdependent mechanisms form the backbone of biofilm resilience [27]:

- Physical barrier – the biofilm matrix impairs antibiotic penetration.
- Microenvironmental protection – nutrient gradients, acidic pH, hypoxia, and waste accumulation suppress antibiotic efficacy.
- Persistence – dormant subpopulations (“persister cells”) evade killing due to metabolic inactivity.

This resistance is further amplified by biochemical defences (extracellular polysaccharides, eDNA, antibiotic-degrading enzymes, efflux pumps), molecular adaptations (horizontal gene transfer, mutational plasticity), and host–pathogen interactions (sub-inhibitory drug exposure, oxidative stress, QS) [27]. Collectively, these layered defences drive chronic infection and therapeutic failure in HAI.

#### Why conventional antibiotics fail against biofilms

Despite being genetically identical, bacteria within biofilms exhibit markedly reduced susceptibility to antibiotics compared to planktonic cells. This is not primarily due to classical resistance mechanisms but results from the structural, physiological, and metabolic heterogeneity of the biofilm state [19].

The EPS matrix acts as a diffusion barrier, limiting antibiotic penetration, particularly for aminoglycosides. However, incomplete efficacy cannot be explained by penetration alone [19]. Microenvironmental gradients of oxygen, nutrients, and pH create metabolically inactive zones, where bacteria become intrinsically tolerant to antibiotics targeting active cellular processes. In addition, biofilms contain persister cells – dormant, non-replicative variants that survive antibiotic exposure without genetic resistance and regain susceptibility upon regrowth [19].

However, most of these mechanisms have been predominantly demonstrated in *in vitro* models, and their relative contribution in complex *in vivo *biofilm-associated infections remains incompletely understood [28], [29]. In clinical settings, this multifactorial tolerance contributes to persistent DAI, often necessitating device removal and prolonged combination antimicrobial therapy, thereby increasing healthcare costs and patient morbidity [30], [31].

#### Why conventional antifungals fail against biofilms

Fungal biofilms, particularly those of Candida species, show markedly increased resistance to antifungals compared to planktonic cells [26]. This is due to a protective extracellular matrix, metabolic dormancy, and the presence of persister cells. QS and high cell density further alter antifungal susceptibility [26]. These adaptive, non-genetic mechanisms render conventional therapies largely ineffective. In clinical settings, Candida biofilms frequently exist as part of polymicrobial communities, particularly with *Staphylococcus (S.) aureus*, where cross-kingdom interactions enhance biofilm robustness, antifungal tolerance, and virulence [32], [33].

#### Mechanisms of tolerance and “persister” cells

Biofilm-associated antibiotic tolerance arises predominantly from non-genetic, physiological adaptations rather than conventional resistance mechanisms. The biofilm’s ECM limits antimicrobial penetration, while nutrient gradients and microenvironmental stressors create zones of low metabolic activity that reduce antibiotic efficacy [22], [34], [35]. Additionally, changes in cellular physiology, such as slowed growth, altered redox states, and stress response activation, further impair antibiotic action. These features collectively confer a reversible, phenotypic tolerance that resolves upon biofilm dispersion. In addition to these innate defenses, a critical subpopulation of cells, termed persisters, enter a dormant, non-replicative state that renders them transiently tolerant to antibiotics [22], [34], [35]. Unlike genetically resistant strains, persister cells do not harbor resistance mutations and regain susceptibility upon regrowth. However, their survival under antimicrobial pressure enables recurrence and may facilitate the eventual emergence of resistant mutants [22], [34], [35]. Together, these mechanisms make biofilm infections uniquely refractory to treatment.

### Clinically relevant biofilm-forming pathogens

Most commonly associated biofilm-forming microorganisms linked with device-associated infections are *S. aureus, S. epidermidis, S. capitis, S. lugdunensis, Enterococcus (E.) faecalis, E. coli, Klebsiella (K.) pneumoniae, Enterobacter* spp., *Proteus (P.) mirabilis, Pseudomonas (P.) aeruginosa, Candida (C.) auris, C. albicans *and *C. rugosa* [19], [36], [37], [38], [39], [40].

### Where biofilms thrive – clinical hotspots and devices

An estimated 65% of microbial infections and up to 80% of chronic infections are linked to biofilm formation. These highly structured microbial communities colonize both host tissues and indwelling medical devices, including prosthetic implants, CVCs, urinary catheters, and ET – as well as hospital environmental reservoirs such as sinks, tubing, and surfaces [41]. In clinical environments, biofilms act as persistent reservoirs for nosocomial pathogens, driving AMR and fuelling recurrent infections despite targeted therapy [42].

#### Common device-associated niches

Medical devices serve as prime substrates for biofilm development, transforming life-saving interventions into persistent infection reservoirs. Biofilm-associated infections are central to the burden of CLABSI, CAUTI, VAP and SSI, particularly those linked to prosthetic implants.

CVCs are major sites of biofilm formation and account for a significant proportion of CLABSI [43]. Biofilms form rapidly on catheter surfaces, with short-term use (<10 days) associated with extraluminal colonization and long-term use (>30 days) favouring intraluminal biofilm development [44]. Common pathogens include *S. aureus, S. epidermidis, E. faecalis, K. pneumoniae, P. aeruginosa*, and *C. albicans* [38]. These biofilms evade host immunity and antimicrobial therapy, often leading to septicemia and complications such as endocarditis, making prevention through aseptic techniques and antimicrobial-impregnated catheters essential [45].

The ET play a central role in VAP by impairing mucociliary clearance, suppressing cough reflexes, and facilitating entry of oropharyngeal flora into the lower airways [46], [47]. Biofilms forming on the internal lumen act as persistent reservoirs that protect pathogens from antibiotics and host defenses, contributing to relapse and prolonged infection [48], [49]. VAP is predominantly caused by highly virulent and multidrug-resistant ESKAPE pathogens: *E. faecium, S. aureus, K. pneumoniae, A. baumannii, P. aeruginosa*, and *Enterobacter* spp. – with *E. coli* increasingly implicated [50]. Despite advances in infection control practices, complete eradication of device-associated biofilms remains challenging, and clinical management often relies on device removal rather than pharmacological eradication alone [51].

Urinary catheters are a key risk factor for CAUTI, with most long-term catheterized patients developing bacteriuria [52]. Biofilms rapidly form on catheter surfaces, facilitating ascending infection by pathogens such as *E. coli, P.s mirabilis, P. aeruginosa, E. faecalis*, and *S. epidermidis*, often in polymicrobial communities [53]. These biofilms can obstruct urinary flow, induce inflammation, and predispose to urosepsis, highlighting the importance of minimizing catheter duration, maintaining closed systems, and using antimicrobial-coated devices [52].

Orthopaedic implants are highly prone to biofilm-associated SSIs, which may present as acute, chronic, or hematogenous infections [54]. Biofilm-embedded pathogens, including* S. aureus* (including MRSA), *S. epidermidis, E. faecalis, P. aeruginosa*, and *K. pneumoniae*, form polymicrobial consortia that resist host defences and antibiotic therapy [54], [55]. Persistent infection leads to chronic inflammation, osteolysis, implant loosening, and prosthetic failure, necessitating preventive strategies including perioperative prophylaxis, strict asepsis, and development of anti-biofilm implant surfaces [41], [56], [57].

### Device surface determinants of biofilm formation

Surface roughness, material composition, and interfacial physicochemical properties are not passive features but key drivers of microbial adhesion and biofilm maturation. Increased surface roughness promotes microbial retention by expanding surface area and creating protective niches that reduce shear-mediated detachment, leading to greater early biofilm accumulation on rougher implant surfaces [58]. Beyond topography, material composition and surface chemistry, such as surface free energy, hydrophobicity, and charge, govern the strength and specificity of bacterial attachment, with hydrophobic interactions often favoring pathogen adherence [59]. Additionally, differences in biomaterials (e.g., titanium, zirconia, polymers) and their surface modifications significantly influence biofilm burden, highlighting the absence of a universally biofilm-resistant material [60]. Following implantation, rapid conditioning by host proteins like fibrinogen and fibronectin further enhances microbial colonization [59]. Collectively, these factors highlight how device-related properties shape microbial colonization, directly defining where biofilms thrive.

### Biofilms as hidden drivers of healthcare-associated infections

#### Diagnostic blind spots – why biofilms escape detection

Despite their critical role in DAI, biofilms remain diagnostically elusive due to fundamental limitations in current detection methods as shown in Table 1 (Tab. 1) [61]. Standard cultures may yield false negatives due to low metabolic activity and poor recovery of biofilm-embedded organisms; moreover, even when positive, they detect only planktonic cells and fail to identify the sessile biofilm phenotype, including EPS matrix and altered physiology [61], [62], [63]. Routine imaging similarly lacks sensitivity for detecting biofilms on indwelling devices such as catheters, prostheses, and ETs. These limitations contribute to underrecognition of biofilm-mediated infections and may explain the discordance between in vitro susceptibility results and clinical outcomes [61], [62], [63]. Although standardized in vitro models (e.g., American Society for Testing and Materials) exist, they fail to replicate the dynamic and heterogeneous nature of in vivo biofilms, and laboratory strains may lose biofilm-forming capacity over time [64]. Emerging approaches, including experimental biomarkers and artificial intelligence, show promise for early non-invasive detection; however, the lack of validated biofilm-specific biomarkers remains a major limitation [65], [66], [67], [68], [69], [70], [71], [72]. While advanced molecular and imaging techniques – such as 16S rRNA sequencing, next-generation sequencing, sonication, Confocal Microscopy, cryo-SEM, and atomic force microscopy – provide improved insights, their limited scalability restricts routine clinical use, leaving a persistent gap in the early and accurate diagnosis of biofilm-associated infections [73], [74], [75], [76], [77], [78], [79].

#### Epidemiology and clinical burden of biofilm-associated HAIs

Biofilm-associated infections account for up to 80% of all bacterial infections globally, with 60–70% of nosocomial infections directly linked to biofilm formation [80], [81], [82]. WHO and Centers for Disease Control and Prevention surveillance highlight increasing prevalence in ICU settings, where biofilm-forming pathogens like MRSA and *E. coli* exhibit MDR rates ranging from 17.9% to 100% [83]. The burden is amplified in patients with diabetes, chronic kidney disease, and multiple comorbidities, where biofilm-driven infections prolong ICU stay, escalate treatment costs, and increase mortality [2], [83].

#### Therapeutic roadblocks: why conventional therapy fails

Empirical monotherapy, a mainstay of early HAI management, fails against biofilm-embedded pathogens due to their fortified multicellular architecture [84]. Encased in a dense extracellular matrix, these bacteria exhibit up to 1,000-fold increased antibiotic tolerance via impaired drug penetration, metabolic dormancy, and phenotypic persistence [85]. Furthermore, biofilms act as genetic reservoirs for horizontal gene transfer, accelerating the spread of antimicrobial resistance across hospital ecosystems [86]. These adaptive mechanisms collectively render monotherapy ineffective – driving chronicity, relapse, and therapeutic failure, thus mandating a shift toward precision diagnostics and multi-targeted interventions [87].

#### Sub-inhibitory antibiotic exposure – a hidden catalyst of biofilm growth

Paradoxically, antibiotics may aggravate biofilm-associated infections [88]. Sub-inhibitory concentrations (sub-MICs), commonly encountered in poorly perfused tissues or between dosing intervals, not only facilitates biofilm maturation but can also modulate virulence, surface properties, and gene expression [89]. Notably, cell wall-active agents such as ampicillin and vancomycin induce envelope stress and enhance extracellular DNA (eDNA) release, reinforcing biofilm architecture [90]. Moreover, sub-MIC exposure can drive genetic adaptation through increased mutation, recombination, and horizontal gene transfer, thereby facilitating persistence and antimicrobial resistance [91].

## Discussion

### Anti-biofilm preventive strategies in clinical use

#### Antibacterial and antifouling coatings

Surface coatings incorporating antibiotics, antiseptics, hydrophilic polymers, silver nanoparticles, and antimicrobial peptides inhibit initial microbial adhesion and early biofilm formation [92]. Polihexanide covalently bound to a titanium alloy is bactericidal against both Gram-positive and Gram-negative bacteria. Modelling suggests that concentrations effective for antiseptic purposes are maintained near the surface for periods exceeding 6 months. Crucially, the polyhexanide film has no adverse effects on MG63 cells within a 48-hour cell culture. Across all measurement time points, viability and proliferation in the uncoated control group typically ranged from ≥90–95% with unchanged collagen synthesis. Within 15 minutes, the initial adhesion and spreading of osteoblasts on the test specimens were promoted [93], [94], [95], [96], [97]. Nanomaterials, with high surface-to-volume ratio and tunable physicochemical properties, disrupt biofilm structure, enhance antibiotic penetration, and reduce resistance development [98], [99]. These coatings act via release-based or contact-killing mechanisms while also preventing protein and cellular deposition critical for biofilm initiation [100], [101], [102]. Nanocoatings (e.g., nanosilver, titanium, copper, zinc oxide) show promise but face challenges in biocompatibility and long-term safety [103], [104]. Advances in lipid-coated nanoparticles, polymer-functionalized surfaces, and hydrogel or biodegradable alloy coatings have improved drug delivery and surface compatibility [38], [105], [106], [107]. Recent advances in cell membrane-coated nanocarriers illustrate the versatility of lipid nanotechnology in enhancing implant safety and performance [108]. Emerging systems including cell membrane-coated nanocarriers, enzyme-functionalized nanoparticles, and biofilm-responsive platforms, enable targeted EPS disruption and improved efficacy against polymicrobial biofilms, although approaches like nanoscale bacterial debridement remain experimental [109], [110], [111]. Emerging approaches like “nanoscale bacterial debridement”, selectively detaching bacteria from biofilms, hold future potential but require further investigation [112]. 

#### Surface engineering to resist adhesion

Antiadhesive surfaces reduce bacterial-surface interactions, allowing microorganisms to be easily removed before biofilm maturation [113]. Techniques include anchoring polymer brushes to device surfaces using barnacle cement or polydopamine [114]. Such modifications on stainless steel have shown reduced protein adsorption and robust stability [115]. Tannic acid–based bifunctional coatings further enhance biofouling resistance [116].

#### Antimicrobial hydrogel and alloy coatings

Hydrogel coatings and antibacterial surface modifications have demonstrated efficacy in preventing orthopedic implant-associated infections [117], [118]. In parallel, biodegradable metal alloys with intrinsic antimicrobial properties are emerging as viable alternatives to conventional implants [119]. These innovations reduce microbial colonization and offer promise for clinical translation pending further validation.

#### Surface modification of biomaterials

Modifying implant surface properties (e.g., energy, roughness, hydrophilicity) without coatings has shown to reduce bacterial adhesion [120]. By altering surface characteristics, these strategies prevent bacterial settlement and biofilm initiation [121]. Techniques like pulsed laser evaporation help engineer inherently anti-adhesive surfaces.

#### Natural products in biofilm control: emerging strategies beyond antibiotics

Natural products inhibit biofilms by targeting adhesion, QS, and maturation [7]. Plant-derived compounds (flavonoids, terpenoids, and phenolics) disrupt biofilms by inhibiting surface attachment and QS without direct bactericidal action [122].

Phytochemicals like emodin, curcumin, and citrus flavonoids reduce virulence and biofilm integrity [123]. Natural agents like honey and cranberry proanthocyanidins further reduce mature biofilm biomass [124], [125]. Probiotic strains, especially *Lactobacillus* spp., further inhibit adhesion and destabilize biofilms via bacteriocins and biosurfactants [19].

#### Next-generation antimicrobials (NGAs) – disrupting medical device biofilms

NGAs combat medical device biofilms through multi-targeted mechanisms that overcome AMR [126]. Enzymatic agents such as DNase I and proteases (e.g., Proteinase K, dispersin B) degrade EPS components, promoting biofilm dispersal and enhancing antibiotic penetration [127], [128], [129], [130]. Anti-adhesion strategies (pilicides, glycomimetic) prevent initial bacterial attachment while while QS inhibitors disrupt biofilm maturation. Additionally, cyclic di-GMP modulators and nitric oxide donors induce biofilm dispersal via intracellular signaling pathways [126], [131], [132], [133].

#### Quorum sensing inhibition and biofilm disruption

Targeting QS pathways with small molecules, enzymes (e.g., DNase I), or natural extracts (e.g., rosmarinic acid, ginger) can prevent biofilm formation [134], [135]. These methods interfere with bacterial communication and matrix integrity, thereby impairing biofilm stability and maturation [121]. NSAIDs (e.g., meloxicam, aspirin) and antibiotics (e.g., azithromycin, ciprofloxacin) exhibit QS inhibitors activity [136]. Synergistic combinations, such as resveratrol with aminoglycosides, enhance biofilm disruption and prevent QS inhibitors resistance [137]. Adjuncts like D-amino acids and proteases enhance antibiotic efficacy against resistant biofilms [138], [139]. 

#### Plant-based strategies for control of biofilm associated infections

Phytochemicals (phenolics, flavonoids, terpenoids, alkaloids, essential oils) act via QS inhibition, membrane disruption, EPS degradation, and nutrient sequestration. They are eco-friendly, often synergistic, and less prone to resistance development [140].

Examples include *Hypericum lydium* extracts (anti-MRSA activity), *Cochlospermum regium* (phenol-rich) against MRSA biofilms, *Persea americana* seed extracts (enhanced wound healing and antibiofilm effect), isoflavonoid- and xanthone-rich *Iris pseudacorus, Curcuma aromatica* flavonoids/alkaloids for Gram-positive biofilms and *Frangula angus* and *Hymenocallis littoralis* with broad-spectrum antibiofilm activity.

#### Nanotechnology-based strategies for control of biofilm associated infections

Nanoparticles (NPs) offer high surface reactivity, biofilm penetration, and multi-mechanistic antimicrobial action (membrane disruption, ROS generation, DNA damage) [140]. Examples are:

- Silver NPs (AgNPs) are effective suture/device coatings with broad-spectrum action; TiO_2_–Ag hybrids zwitterionic AgNP dressings works by ROS generation (TiO_2_) with AgNP bactericidal action [140].
- Composite and functionalized NPs such as amphora-shaped porous Ti implants deters bacterial attachment, CaP–Ag coatings prevent adhesion on bone implants and release bactericidal Ag^+^ for orthopedic devices [140].
- Other nanomaterials are polymeric NPs, dendrimers, and liposomes for targeted delivery and improved biofilm penetration [140]

However, translation into clinical practice remains limited due to concerns regarding long-term toxicity, scalability, regulatory approval, and cost-effectiveness, particularly in low-resource healthcare settings [141].

### Established and clinically applicable therapeutic anti-biofilm approaches

#### Physical chemical and biological approaches

Physical methods such as ionizing/UV radiation and ultrasonic cavitation disrupt biofilms through mechanical and oxidative stress [142]. Cold atmospheric plasma enhances eradication through reactive oxygen species (ROS)/reactive nitrogen species, mediated damage, with synergistic effects when combined with ultrasound [143]. Microneedles further improve antimicrobial penetration by breaching the EPS matrix [144]. In contrast, chemical agents are limited by toxicity, instability, and resistance, whereas biological strategies, such as enzymes, and bacteriophages, offer greater specificity and safety [145].

#### Bacteriocins

These are ribosomally synthesized antimicrobial peptides (AMPs), have emerged as promising antibiofilm agents with multifaceted mechanisms [146], [147]. They inhibit bacterial adhesion and biofilm formation, reduce EPS production, and disrupt mature biofilms [146], [148]. Studies demonstrate significant reductions in biofilm biomass and matrix integrity across key pathogens, including *S. aureus, P. aeruginosa*, and *E. faecalis* [149]. Additionally, bacteriocins can eradicate biofilm-associated cells, highlighting their potential in managing biofilm-mediated healthcare-associated infections [150].

#### Conventional management of biofilm-associated infections

Biofilm-associated infections require combined surgical and antimicrobial management. Superficial infections may respond to drainage, whereas deep or device-related infections often require debridement, prosthesis removal, and targeted therapy [151]. Negative pressure wound therapy augments debridement by promoting granulation and reducing bioburden [152]. Antibiotic-loaded spacers (e.g., vancomycin or gentamicin) with rifampin-fluoroquinolone regimens improve eradication of staphylococcal biofilms, whereas resistant infections may require agents such as linezolid, daptomycin, tigecycline, or carbapenems [140]. Polymicrobial biofilms further complicate treatment by accelerating tissue damage and reducing antibiotic efficacy [153].

#### Antimicrobial peptides (AMPs)

AMPs disrupt bacterial membranes, suppress biofilm-related gene expression, and modulate QS, offering potent activity against both planktonic and biofilm-embedded pathogens [154]. Peptides like LL-37 and lactoferrin derivatives exhibit broad-spectrum antibiofilm effects, particularly in respiratory infections, and synergize with antibiotics to enhance efficacy [155], [156]. Lactoferrin, an innate immune component, prevents *P. aeruginosa* biofilm formation by stimulating bacterial twitching motility [10]. Metal-binding AMPs, such as Gaduscidin-1, are effective in hostile microenvironments, including those seen in *P. aeruginosa* biofilms [157], [158]. 

### Established biological therapies

#### Bacteriophage therapy

 Bacteriophages disrupt biofilms through enzymatic degradation (e.g., haemolysinase, depolymerases) and lytic replication, effectively targeting both antibiotic-sensitive and resistant bacteria [10], [159], [160]. Specific phages (e.g., Φ15, Φ29, PD1, PE2, T4) have demonstrated efficacy against Pseudomonas, Staphylococcus, Salmonella, and Klebsiella biofilms [161], [162]. Phage-antibiotic combinations (e.g., T4 with tobramycin, or phage with amoxicillin) enhance biofilm eradication and limit resistance emergence [163]. Phage cocktails further expand host range and reduce resistance development [164].

#### Enzyme-based disruption of bacterial biofilms

Enzymes (oxidases, proteases, and polysaccharide-degrading hydrolases) disrupt biofilms by degrading EPS, interfering with QS, and inhibiting maturation [164], [165]. Their high specificity makes them ideal biological anti-biofilm agents. However, their stability is limited; immobilization strategies, such as crosslinked enzyme aggregates (CLEA), enhance stability and reusability [166]. Magnetic CLEA formulation (m-combi-CLEA) have shown >75% inhibition of *E. coli and* *S. aureus* biofilms, highlighting their therapeutic potential [167]. 

#### Vaccines

Vaccines incorporating biofilm-derived antigens represent a promising strategy to enhance protection against persistent infections [168]. In *Bordetella pertussis*, biofilm-derived outer membrane vesicles induce stronger immunogenicity and protection than planktonic counterparts, including against pertactin-deficient strains, and generate durable mucosal CD4^+^ memory responses [169], [170]. While still under investigation, biofilm-based vaccines offer a compelling direction for future anti-biofilm immunotherapies [14].

#### Monoclonal antibodies as therapeutics for fungal biofilm infections

Monoclonal antibodies (MAb) targeting biofilm-specific antigens enable radioimmunotherapy through MAb-guided alpha radiation, allowing in situ treatment when device removal is contraindicated [171]. Prophylactic MAb administration prevents Cryptococcal biofilm establishment post-implantation [172]. Chitosan device coatings provide biocompatible protection by disrupting microbial membrane integrity and preventing surface colonization [173]. *In vivo* studies demonstrate efficacy against *Candida* biofilms on CVCs without host cell toxicity [174]. Optimized antifungal approaches – targeting early biofilm stages, novel formulations (amphotericin B lipid complex), and combination therapies – significantly enhance treatment efficacy compared to conventional methods [175].

### Emerging and advanced therapeutic strategies

The growing threat of antimicrobial resistance underscores the need for novel approaches. 

#### Energy-based methods

Electric fields (e.g., DC, AC, pulsed electric fields) induce membrane disruption and enhance antibiotic uptake through a “bioelectric effect” [176], [177]. These approaches help destabilize the biofilm matrix and enhance drug penetration into biofilm-embedded bacteria [121]. In vitro and animal studies show synergy with antibiotics, reducing biofilm burden, but further clinical validation is needed [178].

Low-frequency ultrasound enhances antibiotic penetration and disrupts EPS matrices. Devices delivering surface acoustic waves weakens biofilm cohesion and promotes antimicrobial access and have demonstrated >85% reduction in common pathogens’ biofilms when combined with antimicrobials [179]. Although ultrasound enhances drug penetration, its clinical applicability is limited by optimization challenges, device standardization, and gaps in clinical translation [180].

Antimicrobial photodynamic therapy (aPDT) leverages light-activated photosensitizers to generate ROS and directly damages biofilm components and microbial cells, leading to effective biofilm disruption [121]. Laser and LED-based systems (405–940 nm) have successfully eradicated biofilms on various medical substrates. Clinical use is constrained by limited light penetration, challenges in effective photosensitizer delivery, and difficulty in treating deep-seated infections, with further safety data required for clinical translation [181], [182], [183].

#### Molecular and genetic tools

CRISPR/Cas systems enable precise disruption of biofilm formation by targeting key regulatory genes, such as *icaA* in *S. aureus*, *lasR/rhlR* in *P. aeruginosa*, and *pelA* in *P. aeruginosa* and *E. coli*, thereby reducing biofilm biomass and adhesion [184], [185], [186], [187]. Additionally, CRISPR-associated nucleases can selectively degrade AMR determinants, re-sensitizing biofilm-embedded pathogens to conventional antibiotics [188]. To overcome delivery barriers, engineered bacteriophages carrying CRISPR constructs have shown efficacy against* K. pneumoniae* biofilms, while nanocarrier and liposomal systems further enhance penetration and therapeutic efficiency within the biofilm matrix [189]. Clinical translation is hindered by delivery barriers, off-target effects, biosafety concerns, and complex regulatory approval pathways [190].

Aptamers, synthetic single-stranded oligonucleotides or peptides, exhibit high-affinity, target-specific binding via defined 3D structures [191]. They disrupt biofilms by depolarizing bacterial membranes and enhancing antibiotic delivery. Aptamer-functionalized nanomaterials, such as aptamer-graphene oxide complexes, have shown >90% inhibition of *Salmonella typhimurium* biofilms [192]. Their therapeutic application is limited by rapid degradation *in vivo*, poor stability, and challenges in targeted delivery and large-scale production [193].

Peptide nucleic acids (PNAs) bind bacterial DNA with high specificity and affinity, showing promise against MDR pathogens and biofilms [194]. Although limited by poor penetration, delivery strategies such as conjugation with cell-penetrating peptides enhance efficacy. PNAs targeting *ftsZ, efaA*, or *acpP* genes inhibit bacterial division and biofilm formation in *Escherichia coli, Enterococcus* spp., and *Hemophilus influenzae* [195], [196], [197]. Synergistic combinations with antibiotics (e.g., polymyxin B) potentiate anti-biofilm effects, making PNAs a compelling NGA strategy [198]. PNAs face significant barriers including poor cellular uptake, delivery challenges, and potential toxicity at higher concentrations [199].

Real-time biofilm detection via biosensors (e.g., impedance-based systems, Raman spectroscopy, qPCR) allows for early identification enabling timely therapeutic intervention before biofilm maturation, improving treatment outcomes [121], [200], [201]. Techniques like SERS and interdigitated microelectrodes offer high sensitivity for detecting pathogens on medical surfaces [121]. Despite high sensitivity, widespread clinical use is limited by high cost, need for specialized infrastructure, and challenges in real-time clinical integration [180].

#### Targeted molecular anti-biofilm strategies

Catabolite control protein A (CcpA) is a key regulator of biofilm formation in *S. aureus*, promoting adhesin and eDNA production (cidA/icaA) while repressing the sak gene, which encodes staphylokinase and enhancing virulence via a-hemolysin [202]. Inhibiting CcpA–DNA binding reduces toxin expression against *S. aureus* biofilm-related infections, highlighting the CcpA–Sak axis as a promising low-toxicity therapeutic potential [203], [204].

Functional amyloids play a central role in biofilm development across several bacterial species [10]. Small molecules such as FN075 and BibC6 in *E. coli*, and AA-861 or parthenolide in *Bacillus subtilis*, inhibit amyloid fiber formation or disrupt established biofilms, thereby reducing virulence [205], [206]. These findings highlight that targeting amyloid assembly can effectively weaken biofilm structure and persistence. Table 2 (Tab. 2) summarizes all biofilm-eradicating agents and their action.

### Clinical translation and real-world challenges

Although all these strategies demonstrate significant antibiofilm activity in vitro and in preclinical models, robust clinical evidence supporting their routine use remains limited, highlighting a critical gap between experimental innovation and bedside application [207], [208]. Most approaches, including NPs, AMP, QS inhibitors, and enzymatic therapies, are supported primarily by in vitro or animal model data, with a paucity of large-scale randomized clinical trials [209], [210]. Current management of biofilm-associated HAIs continues to rely heavily on device removal, prolonged antimicrobial therapy, and infection control measures [51].

Cost, scalability, toxicity, and regulatory barriers further hinder clinical adoption, particularly in low- and middle-income countries where the burden of HAIs is highest [141], [211]. Even promising interventions such as antimicrobial coatings and lock therapies have demonstrated variable efficacy in clinical settings. Bridging this gap requires well-designed clinical trials, standardized evaluation models, and integration of biofilm-specific strategies into existing infection control frameworks [212].

## Conclusion

Biofilms are a critical yet often underrecognized driver of HAIs, contributing to persistence, recurrence, and antimicrobial resistance. Through coordinated processes such as adhesion, extracellular matrix formation, and quorum sensing, biofilm-embedded pathogens evade host defenses and exhibit marked tolerance to conventional antimicrobial therapies. As a result, standard treatment strategies designed for planktonic organisms are frequently ineffective, particularly in device-associated infections such as CLABSI, CAUTI, VAP, and SSI.

The intrinsic tolerance conferred by the biofilm matrix, metabolic heterogeneity, and persister cell populations necessitates a paradigm shift toward biofilm-targeted prevention, early detection, and multi-modal treatment approaches. Preventive strategies particularly surface engineering, antimicrobial coatings, and quorum sensing inhibition, offer the most effective opportunity to reduce biofilm establishment, especially in device-associated settings.

Therapeutically, while established approaches such as surgical debridement, device removal, and combination antimicrobial therapy remain the clinical cornerstone, a broad spectrum of emerging interventions, including nanotechnology-based systems, antimicrobial peptides, bacteriophages, enzyme-based matrix disruption, and molecular tools such as CRISPR – demonstrate promising anti-biofilm activity. However, a critical translational gap persists, as most of these strategies remain confined to in vitro and preclinical models, with limited validation in large-scale clinical trials.

Importantly, this review underscores the need for integration of biofilm-specific diagnostics, preventive strategies, and targeted therapeutics into existing infection control frameworks. Advancing the field will require standardized models for biofilm evaluation, robust clinical studies, and interdisciplinary collaboration bridging microbiology, material science, and clinical medicine.

Addressing biofilm-associated HAIs demands a shift from reactive treatment to proactive prevention and precision-targeted therapy. Bridging the gap between mechanistic understanding and clinical application is essential to reduce the burden of HAIs, combat AMR, and improve patient outcomes in modern healthcare systems.

## Notes

### Authors’ ORCIDs

- Anand G: https://orcid.org/0009-0008-0473-389X
- Lahariya R: https://orcid.org/0009-0003-5769-4509

### Funding

None. 

### Competing interests

The authors declare that they have no competing interests.

### Generative AI statement

The author(s) declare that generative AI (ChatGPT) was used exclusively to assist with language editing, grammatical refinement, and improvement of clarity of expression. The author(s) reviewed and edited the manuscript and take full responsibility for its content. The figures were generated using Google’s generative AI tools based on original text developed by the authors. The generated images were created specifically for this manuscript and are not reproduced from any previously published source.

## Figures and Tables

**Table 1:**
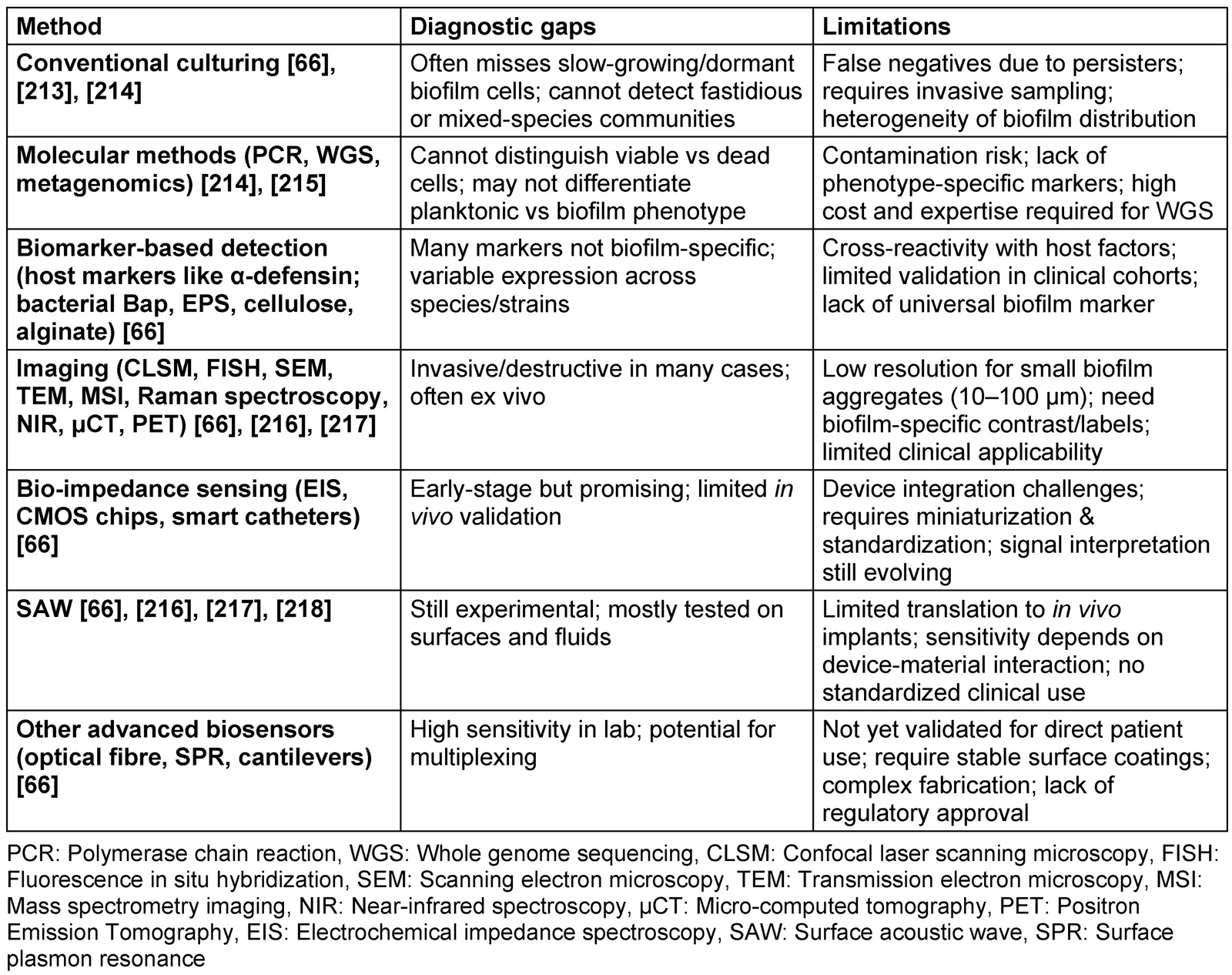
Diagnostic gaps and limitations of biofilm detection methods

**Table 2:**
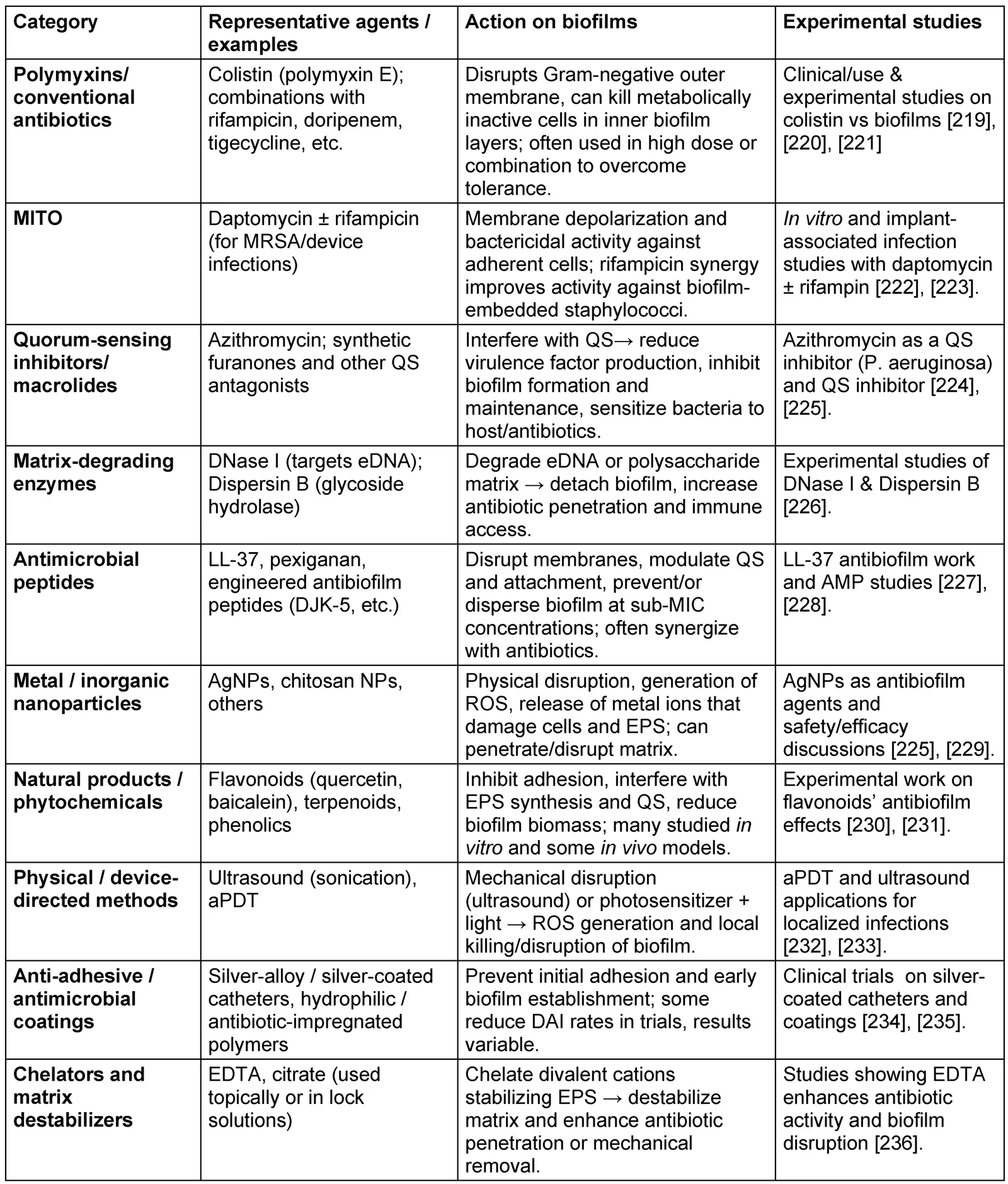
Biofilm-eradicating agents with their action

**Figure 1:**
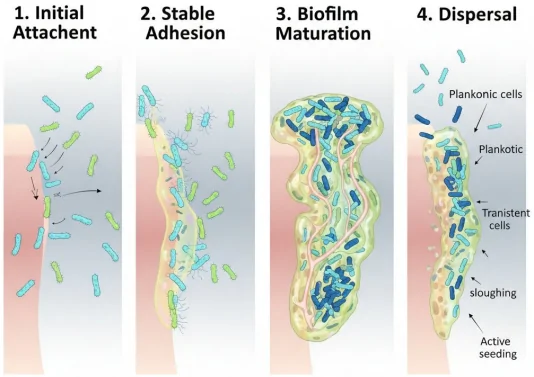
Formation and maturation of bacterial biofilm

**Figure 2:**
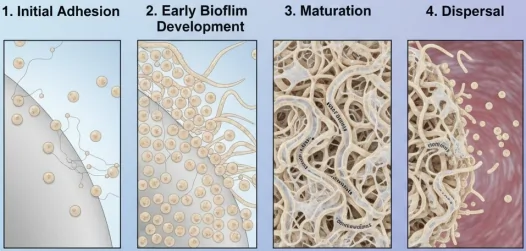
Formation and maturation of fungal biofilm
